# Percutaneous thermal ablation combined with transcatheter arterial chemoembolization for hepatitis C virus-related hepatocellular carcinoma: Efficacy and survival

**DOI:** 10.3389/fonc.2022.978614

**Published:** 2022-09-23

**Authors:** Yu Sun, Honghai Zhang, Jiang Long, Yonghong Zhang, Jiasheng Zheng, Chunwang Yuan

**Affiliations:** Liver Disease and Cancer Interventional Therapy Center, Beijing Youan Hospital, Capital Medical University, Beijing, China

**Keywords:** hepatocellular carcinoma, transcatheter arterial chemoembolization, percutaneous thermal ablation, overall survival, recurrence, prognosis

## Abstract

**Objective:**

The aim of this study was to investigate the efficacy and survival of Hepatitis C virus (HCV) -related hepatocellular carcinoma (HCC) undergoing percutaneous thermal ablation combined with transcatheter arterial chemoembolization (TACE).

**Methods:**

A total of 83 HCV-related HCC patients who were treated with percutaneous thermal ablation combined with TACE were retrospectively analyzed. The demographic and clinical data were collected. The overall survival (OS) and recurrence free survival (RFS) rates were assessed by the Kaplan-Meier method. Univariate and multivariate Cox regression analysis was used to assess independent risk factors of OS and RFS.

**Results:**

92.8% patients (77/83) and 96.6% (170/176) tumor lesions achieved complete response (CR) 1 month after all treatment, and 10.8% (9/83) patients had minor complications. The median OS was 60 months (95% confidence interval (*CI*)= 48.0-72.0), and the 1-, 2-, 3-, 5- and 10-year cumulative OS rates were 94%, 78.3%, 72.3%, 43.4% and 27.5%, respectively. The cumulative RFS rates at 1-, 2-, 3- and 5-year were 74.7%, 49.3%, 30.7% and 25.3%, respectively. Sex (*HR* =0.529, *P*=0.048), ablation result (*HR*=5.824, *P*=0.000) and Albumin-bilirubin (ALBI) score (*HR*=2.725, *P*=0.011) were independent prognostic factors for OS. Alpha-fetoprotein (AFP) (*HR* =2.360, *P* = 0.005) and tumor number(*HR*=2.786, *P*=0.000) were independent prognostic factors for RFS.

**Conclusions:**

Percutaneous thermal ablation combined with TACE is a safe and effective treatment for HCV-related HCC. Sex, ablation result and ALBI are significant prognostic factors for OS. AFP and tumor number are significant prognostic factors for RFS.

## Introduction

Primary liver cancer is the sixth most common cancer in the world, accounting for the fourth cause of cancer death worldwide in 2018, with about 841000 new cases and 782000 deaths each year (1). Hepatocellular carcinoma (HCC) accounts for about 85-90% of all primary liver cancers (2). Hepatitis virus B Virus (HBV) and Hepatitis C virus (HCV) are the main risk factors for HCC (3, 4). Although the incidence of HCV-related HCC is lower than that of HBV-related HCC, with the aging and population growth, the expected burden of HCV-related HCC in China is rising (5). Curative therapies for early-stage HCC includes surgical resection, liver transplantation and percutaneous ablation. Owing to cirrhosis almost accompanying all HCV-related HCC, percutaneous ablation, especially thermal ablation is usually useful alternative modalities for these patients. Recent studies have showed that percutaneous ablation combined with transcatheter arterial chemoembolization (TACE) may have synergistic effect in the treatment of early and intermediate stages HCC (6). However, there are few studies focused on the efficacy and prognosis of HCV-related HCC receiving percutaneous thermal ablation combined with TACE.

In this study, we aimed to identify the efficacy, safety and survival of HCV-related HCC after percutaneous thermal ablation with TACE in HBV-endemic area.

## Material and methods

### Patients

**Figure 1:**
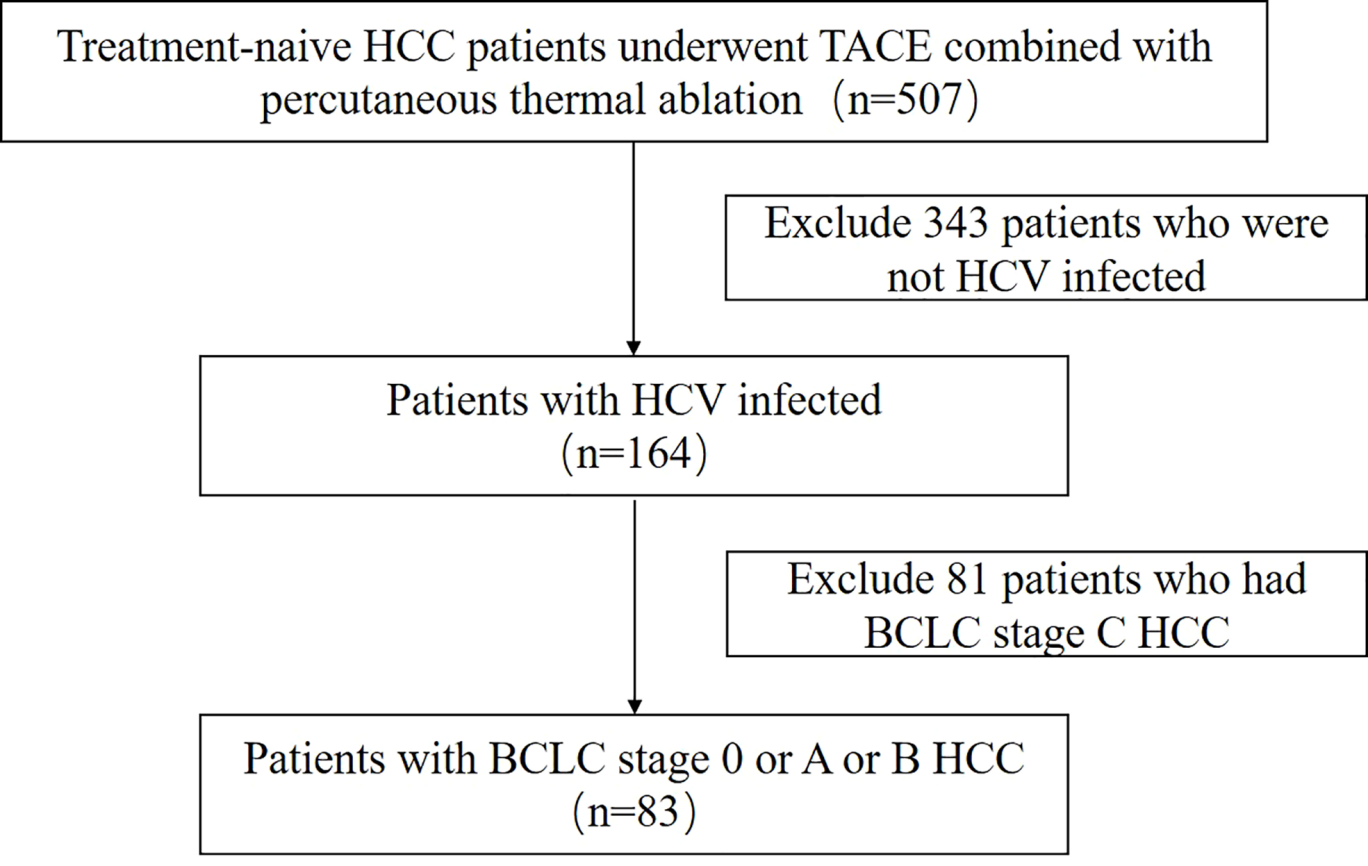
Patient flowchart.

We retrospectively reviewed a total of 507 consecutive treatment-naive patients with HCC who underwent percutaneous thermal ablation combined with TACE at Beijing You An Hospital, Capital Medical University from July 2006 to January 2016. Inclusion criteria for this study were as follows: (1) HCV-infected patients; (2) a single tumor with a maximum size smaller than 7 cm and tumor number less than 5; (3) no invasion of adjacent organs or tumor thrombi in portal, vein and bile ducts system, and no extrahepatic metastasis; (4) no serious non-liver underlying illness including heart, brain, lung, kidney and other organs dysfunction, and no other tumor diseases; (5) liver function of Child-Turcotte-Pugh (CTP) class A and B; ECOG (Eastern Cooperative Oncology Group) performance status score 0~1; (6) platelet count ≥50 × 10^9^/L for percutaneous thermal ablation, prothrombin time ratio ≥ 50% and total bilirubin<50umol/L for both TACE and percutaneous thermal ablation; (7) no upper digestive track bleeding due to portal hypertension within 1 month before TACE; (8) no uncontrolled infection; (9) complete case and follow-up data. This research scheme has been exempted from the requirement of informed consent and approved by the Ethics Committee of our hospital. As summarized in **Figure 1**, among the 507 patients, the remaining 83 patients met the inclusion criteria, including 30 patients who were diagnosed with HCC histologically by liver biopsy, and another 53 patients, who were diagnosed by imaging diagnosis.

### Pretreatment evaluation

The pretreatment assessment of each patient included spiral computed tomography (CT) of chest, either Contrast-enhanced CT (CECT) or contrast-enhanced magnetic resonance imaging (CEMRI) of the abdomen, electrocardiogram, complete blood count (CBC), liver and renal function tests, prothrombin time, alpha-fetoprotein (AFP), HCV RNA. According to the guidelines for diagnosis and treatment of primary liver cancer (China, 2017 edition) (7), patients with maximum tumor diameter 1-2cm confirmed by 2 typical contrast enhancement imaging presentations, or maximum tumor diameter more than 2cm confirmed by 1 typical contrast enhancement imaging presentations or histopathological examinations were diagnosed with HCC.

We collected baseline clinical data including: age, gender, CBC, albumin (ALB), total bilirubin(TBIL), glutamic pyruvic transaminase(ALT), glutamic oxaloacetic transaminase(AST), cholinesterase(CHE), prothrombin time(PT), AFP, CTP grade, tumor characteristics. We calculated Albumin-bilirubin (ALBI) score as follows, ALBI = 0.66 × Log10(TBIL μmol/L) -0.085 ×(ALB g/L). ALBI grade was classified as grade 1 (≤−2.60), grade 2 (−2.60 to −1.39), or grade 3 (>−1.39), respectively.

### TACE

All TACE are conventional-TACE(c-TACE). TACE was performed first.Seldinger catheterization was used to intubate the right femoral artery, and angiography of the hepatic artery was performed to observe the location, size, number, arterial supply of tumors. Catheters and microcatheters (Asahi Intecc Co., Ltd., Japan) was inserted into the target branch. Iodized oil (Guerbet, Villepinte, Seine-Saint-Denis, France), and doxorubicin (Pfizer Inc., NY, USA) suspension emulsion were injected into the arterial branches followed by injection of gelatin sponge particles (350~560um, Hangzhou Alicon Pharmaceutical Technology Co., Ltd. Hangzhou, China). The dose of the drugs depended on the tumor size, liver function, white blood cell count and platelet count of the patient. One week later, CT scan of the abdomen was performed to evaluate the effect of TACE. The TACE procedure was repeated if the effect was not satisfactory.

### Radiofrequency ablation/microwave ablation

Thermal ablation was carried out within 2 weeks after TACE. CT scanning was performed to determine the puncture site and approach. After routine sterilization and focal anesthesia, the RFA electrode needle or microwave antenna was used to puncture the tumor under CT guidance. The ablation was performed according to the predetermined ablation conditions. According to the ablation range of each tumor, the RFA electrode needles or microwave antennas were adjusted to achieve an overlapping ablative margin that would theoretically include the tumor and 0.5~1 cm of surrounding tissue. After the ablation range was satisfied, the electrode needle or microwave antennas were withdrawn, and the needle tunnel was ablated at 70°C-90°C to reduce the risk of hemorrhage or implantation metastasis *via* the needle tunnel. CECT or CEMRI of the abdomen was performed within 1 week after ablation to evaluate technique effectiveness. If the imaging examination showed an enhanced area within or around the original tumor, we suspected that incomplete ablation portions remained, and the RFA/MWA procedure was repeated if the liver function met the requirements.

Treatment was continued until CT or MRI imaging demonstrated necrosis of the entire tumor. CECT or CEMRI was performed one month after the treatment to determine the effects of ablation, which were classified as complete response (CR) or incomplete response (ICR). CR was defined as CECT or CEMRI detection of a non-enhanced area with necrosis at the ablation site of the HCC nodules. Patients with CECT or CEMRI evidence lacking CR were defined as ICR and received repeated salvage RFA/MWA treatment. The evaluations were repeated 1month after salvage treatment. Those who failed to obtain CR after repeated salvage RFA/MWA were regarded as treatment failure (TF). In these cases, liver transplantation, resection, TACE, or other treatments were considered.

### Follow-up

The follow-up protocol included AFP assays, CECT or CEMRI of the abdomen and liver function every 3 months after treatment and more frequently when needed. CT of chest was performed every 6 months or if tumor recurrence was suspected. Tumor recurrence includes local recurrence, intrahepatic recurrence and extrahepatic recurrence. Overall survival (OS) was defined as the interval between the date of the initial TACE and the death, or the end of the study for patients who survived. Recurrence free survival (RFS) was defined as the interval between the date of the CR and the recurrence or death or the end of the study for patients who did not experience recurrence.

### Statistical analysis

SPSS software version 22.0 (SPSS, Inc., Chicago, IL, USA) was used to statistically analyze data. Quantitative variables were expressed as mean ± standard deviation or as medians, ranges. Survival rates were estimated using the Kaplan-Meier method and compared using the log-rank test. The OS and RFS rates were assessed by the Kaplan-Meier method with the log-rank test. Univariate and multivariate analysis was carried out by Cox proportional hazards regression model to assess independent risk factors of OS and recurrence. *P*<0.05 was defined as statistically significant.

## Results

### Characteristics of the patients

Baseline characteristics of the 83 patients who underwent percutaneous thermal ablation combined with TACE were showed in **Table 1**.

**Table 1:** Clinical characteristics of patients.

Variable	Value
Age (years)	
Mean (range)	61.83 ± 8.49 (40-84)
Sex	
Males	49 (59.04%)
Females	34 (40.96%)
Cirrhosis	
No	7 (8.43%)
Yes	76 (91.57%)
Ablation result	
CR	75 (90.36%)
ICR	8 (9.64%)
HCV-RNA	
Positive	69 (83.13%)
Negative	14 (16.87%)
ALBI	
Grade 1	24 (28.92%)
Grade 2	52 (62.65%)
Grade 3	7 (8.43%)
CTP	
A	70 (84.34%)
B	13 (15.66%)
AFP levels(ng/ml)	
≤7	26 (31.33%)
>7	57 (68.67%)
Tumor number	
≤1	37 (44.58%)
>1	46 (55.42%)
Diameter of largest tumor (cm mean (range))	2.77 ± 1.19 (1.00-7.00)
≤3.0	53 (63.86%)
>3.0	30 (36.14%)
Tumor location	
Right lobe of liver	49 (59.04%)
Left lobe of liver	15(18.07%)
Right and left lobe of liver	19(22.89%)
BCLC stage	
0	9 (10.84%)
A	47 (56.63%)
B	27 (32.53%)

SD, standard deviation; HCV, hepatitis C viruses; ALB, albumin; TBIL, total bilirubin; ALT, glutamic pyruvic transaminase; AST, glutamic oxaloacetic transaminase, CHE, cholinesterase; AFP, alpha fetoprotein.

### Treatment response

Among the 83 treated patients, 80 patients underwent a single TACE, and 3 patients underwent two TACE for successful embolization of the tumor artery. We performed 110 thermal ablation including 89 RFA and 21 MWA for 176 tumor lesions in 83 patients. 58 patients underwent a single thermal ablation, 23 patients underwent two thermal ablation and 2 patients were treated with three thermal ablation in order to achieve the complete response. 92.8% patients (77/83) and 96.6% (170/176) tumor lesions achieved CR 1 month after all treatment, and 7.2% (6/83) patients and 3.4% (6/176) tumor lesions were identified as ICR. 100% patients in the BCLC-0 group, 97.9% (46/47) in the BCLC-A group and 81.5% (22/27) in the BCLC-B group achieved CR.

During treatments, there were no serious adverse reactions such as liver failure, biliary bleeding, abdominal bleeding, pericardial tamponade, liver abscess and treatment-related death. 10.8% (9/83) patients had minor complications such as puncture point pain, liver pain, fever, nausea and vomiting, abdominal distension, mild liver function injury, ascites or pleural effusion, and recovered by conservative treatment.

### Follow-up results

**Figure 2:**
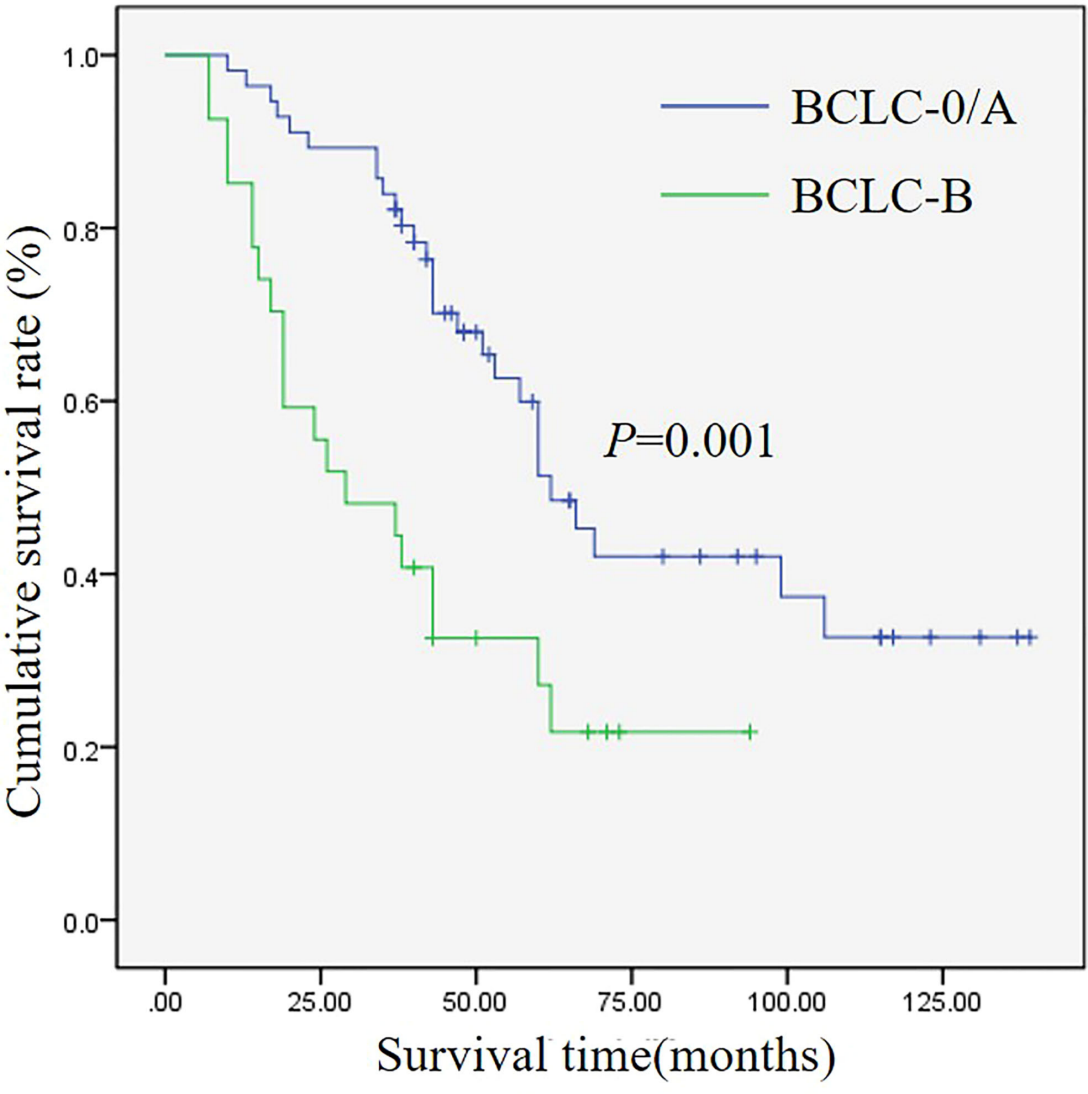
Kaplan-Meier analysis of OS for patients with different BCLC stages.

Until January 31st 2020, the median follow-up period was 73months (ranging from 7-139months). At the end of follow-up, 57.8%(48/83) patients died and 42.2%(35/83) patients survived. The median OS was 60 months (95% confidence interval (*CI*)= 48.0-72.0), and the 1-, 2-, 3-, 5- and 10-year cumulative OS rates were 94%, 78.3%, 72.3%, 43.4% and 27.5%, respectively. The 1-, 2-, 3-, 5- and 10-year cumulative OS rates in patients with BCLC−0/A HCC were 98.2%, 89.3%, 83.9%, 51.4%, 32.7%, and 85.2%, 55.6%, 48.1%, 27.2% in patients with BCLC−B. There was significant difference in OS among the two groups (*χ^2^* = 10.134, *P*=0.001)(**Figure 2**).

During the follow-up period, 77.9%(60/77) patients who achieved CR from the thermal ablation combined with TACE experienced recurrence. The cumulative RFS rates at 1-, 2-, 3- and 5-year were 74.7%, 49.3%, 30.7% and 25.3%, respectively. The median RFS of BCLC BCLC-0/A/B HCC was 32 months (95% *CI*= 23.1-40.9), 28 months (95% *CI*= 18.9-37.1) and 18 months (95% *CI*= 15.9-20.1), respectively.

### Multivariate analysis

The factors associated with OS were summarized in **Table 2**. Univariate analysis

**Table 2:** Univariate and multivariate analysis of overall survival after percutaneous thermal ablation combined with TACE in patients with HCV-related HCC.

Variable		Case No.	Univariate Analysis		Multivariate Analysis	
			HR (95%CI)	P	HR (95%CI)	P
Sex	Males/Females	49/34	0.466 (0.254-0.853)	0.013	0.529 (0.281-0.944)	0.048
Age (years)	<60/≥60	34/49	1.384 (0.767-2.50)	0.281		
cirrhosis	Yes/No	76/7	0.295 (0.071-1.219)	0.092		
HCV-RNA	Positive/Negative	69/14	0.949 (0.421-2.137)	0.899		
Ablation result	CR/ICR	75/8	5.606(2.408-13.052)	0.000	5.824 (2.436-13.926)	0.000
WBC (×10^9^)	<4.0/≥4.0	45/38	1.000 (0.565-1.768)	0.999		
PLT (×10^9^)	<100/≥100	51/32	0.645 (0.352-1.180)	0.155		
ALT(U/L)	<45/≥45	41/42	1.199 (0.676-2.129)	0.535		
AST(U/L)	<37/≥37	17/66	2.047 (0.869-4.820)	0.101		
ALB(g/L)	<35/≥35	30/53	0.551 (0.308-0.986)	0.045		
TBIL (umol/L)	≤21/>21	58/25	1.831 (1.024-3.274)	0.041		
ALBI	Grade 1	24	1		1	
	Grade 2	52	3.035 (1.437-6.409)	0.004	2.725 (1.263-5.881)	0.011
	Grade 3	7	3.859 (1.145-12.998)	0.029	3.059 (0.881-10.617)	0.078
CHE(U/L)	<4000/≥4000	36/47	0.429 (0.242-0.760)	0.004		
PT(s)	≤12.8/>12.8	64/19	1.934 (1.012-3.697)	0.046		
CTP	A/B	70/13	2.712 (1.319-5.580)	0.007		
AFP (ng/ml)	<7/≥7	26/57	1.448 (0.766-2.740)	0.255		
Tumor number	<1/≥1		0.564 (0.312-1.019)	0.058		
Diameter of largest tumor (cm)	≤3/>3	53/30	1.029 (0.569-1.864)	0.924		
Tumor location	Right lobe of liver	49	1			
	Left lobe of liver	15	1.208 (0.549-2.661)	0.639		
	Right and left lobe of liver	19	1.564 (0.803-3.046)	0.188		
Ablation type	RFA/MWA	70/13	1.086 (0.456-2.590)	0.852		

indicated 8 factors were related with OS, and multivariate analysis confirmed that only three factors including sex (*HR* =0.529, *P*= 0.048), ablation result (*HR*=5.824, *P*=0.000), ALBI (*HR*=2.725, *P*=0.011)were independent prognostic factors for OS.

The factors associated with RFS were summarized in **Table 3**. Univariate analysis

**Table 3:** Univariate and multivariate analysis of RFS after percutaneous thermal ablation combined with TACE in patients with HCV-related HCC.

Variable		Case No.	Univariate Analysis		Multivariate Analysis	
			HR(95%CI)	P	HR (95%CI)	P
Sex	Males/Females	44/31	0.492 (0.285-0.849)	0.011		
Age (years)	<60/≥60	31/44	0.964 (0.574-1.616)	0.888		
cirrhosis	Yes/No	68/7	1.454 (0.621-3.401)	0.388		
HCV-RNA	Positive/Negative	61/14	0.872 (0.426-1.783)	0.707		
WBC (×10^9^)	<4.0/≥4.0	41/34	1.639 (0.976-2.753)	0.062		
PLT (×10^9^)	<100/≥100	45/30	1.471 (0.873-2.477)	0.147		
ALT (U/L)	<45/≥45	37/38	1.345 0.805-2.248)	0.258		
AST (U/L)	<37/≥37	15/60	1.130 (0.595-2.148)	0.708		
ALB (g/L)	<35/≥35	25/50	0.889 (0.492-1.606)	0.697		
TBIL (umol/L)	≤21/>21	53/22	0.764 (0.425-1.376)	0.371		
ALBI	Grade 1	23	1			
	Grade 2	48	1.919 (1.052-3.502)	0.034		
	Grade 3	4	1.617 (0.467-5.597)	0.448		
CHE (U/L)	<4000/≥4000	30/45	0.866 (0.500-1.501)	0.608		
PT (s)	≤12.8/>12.8	59/16	0.848 (0.439-1.637)	0.624		
CTP	A/B	66/9	1.386 (0.622-3.087)	0.424		
AFP (ng/ml)	<7/≥7	23/52	2.055 (1.148-3.678)	0.015	2.360 (1.300-4.284)	0.005
Tumor number	<1/≥1	35/40	2.488 (1.446-4.281)	0.001	2.786 (1.599-4.856)	0.000
Diameter of largest tumor (cm)	≤3/>3	53/22	1.258 0.726-2.180)	0.413		
Tumor location	Right lobe of liver	46	1			
	Left lobe of liver	14	0.463 (0.213-1.008)	0.052		
	Right and left lobe of liver	15	0.964 (0.510-1.821)	0.911		
Ablation type	RFA/MWA	63/12	1.333 (0.671-2.647)	0.412		

indicated 4 factors were related with RFS, and multivariate analysis confirmed that two factors including AFP (*HR* =2.360, *P* = 0.005) and tumor number (*HR*=2.786, *P*=0.000) were independent prognostic factors for RFS.

## Discussion

Percutaneous thermal ablation is considered to be the optimum local treatment for patients with early-stage unresectable lesions, liver cirrhosis or elderly patients. Percutaneous MWA had similar therapeutic effects and complication rate compared with RFA for HCC (8). Recent studies revealed that combination of thermal ablation and TACE is an effective option for patients with early or intermedium stage HCC (6, 9). TACE prior to percutaneous ablation can not only block the feeding arteries to reduce tumor burden, but also detect satellite nodules and label range of carcinoma. This treatment mode can increase complete ablation rate and reduce the risk of ablation-related bleeding (10). Zhen et al. (11) divided 189 patients into two groups (RFA group, TACE-RFA group); they found that the 1-, 3-, and 4-year OS for the RFA group and the TACE-RFA group were 85.3%, 59%, and 45.0% and 92.6%, 66.6%, and 61.8%, respectively (*HR*=0.525, 95% *CI*:0.335-0.822, *P*=0.002), and the corresponding RFS were 66.7%, 44.2%, and 38.9% and 79.4%, 60.6%, and 54.8%, respectively(*HR*=0.575, 95% *CI*:0.374 to 0.897, *P* = 0.009).

In this study, the CR rate at 1 month was 96.6% in the combination treatment similar to the obtained in previous studies (12). In subgroup analysis, CR rate of BCLC 0 was 100%, and that of BCLC A was 97.9%. The reason that one patient belonged to BCLC A did not achieve CR was considered that tumors was close to portal vein. In addition, there were only minor complications in 10.8% patients. These data suggest that TACE combined with thermal ablation was safe and effective in the treatment of HCV-related hepatocellular carcinoma, although this is an observational study without control.

It is reported that the long-term prognosis of HCV-related HCC is about 50% with the 5-year OS rate after curative treatment (13). Ren Y et al (9) analyzed 128 HCC patients mainly including HBV-HCC (85.2%) and showed the 1-, 3-, 5- and 8-year survival rates were 90.6%, 76.6%, 68.0%, 68.0%. In our study, after long-term follow-up, the 1-year, 2-year, 3-year, 5-year and 10-year cumulative OS rate were 94%, 78.3%, 72.3%, 43.4% and 27.5%, respectively, and the curative effect in first three years is similar to that reported in HBV-HCC. However, the long term outcome was poorer than that of HBV-HCC reported before. This finding may be attributed to different tumor characteristics or hepatocarcinogenesis between HCV-HCC and HBV-HCC (14, 15).

Previous studies have shown that liver function and field factors might play an important role in prognosis of patients with HCV-related HCC. Due to the subjective judgment of ascites and hepatic encephalopathy, the CTP system was not accurate. Johnson et al. (16) established a novel and objective evaluation model for liver functional reserve assessment called ALBI grade composed of albumin and bilirubin. A number of retrospective studies further confirmed that ALBI grade can predict the prognosis patients with HCC after hepatectomy, liver transplantation, RFA or TACE (17–20). An C et al. (21) recruited 183 patients of HCV-related HCCs and constructed a nomogram which was based on ALBI grade and could provide prediction of long-term outcomes for HCV-related HCC patients after US-PMWA. In the current study, we also identified that ALBI grade determined prognosis for OS of patients with HCV-related HCC underwent thermal ablation combined with TACE. In addition, Univariate and multivariate analysis demonstrated that incomplete ablation of tumors and male were also independent unfavorable prognostic factors for poor OS. Among the 3 factors, incomplete ablation was the most important prognostic factor. Considered reasons of incomplete ablation, except for the fact that it is difficult to achieve CR for tumors close to the large vessels, it may be related to poor differentiation of tumor or microvascular invasion in some patients before therapy. It is suggested to enlarge the cohort and collect the pathological results. The decreased expression of estrogen receptor alfa (ERα) in male patients may explain the worse prognosis of HCV-related cirrhosis and HCC in men than in women (22).

Several studies showed that sustained virological response (SVR) is associated with the favorable long-term survival after curative resection or ablation (23, 24). However, in this study, HCV-RNA is not related to OS of patients after thermal ablation combined with TACE. We consider that there were only few cases (14/69) achieve virological response when antiviral therapy was applied.

The 1-year, 2-year, 3-year and 5-year RFS rates of patients in this study were 74.7%, 49.3%, 30.7% and 25.3%, respectively, similar to those previously reported (25). It is reported that, tumor‐related factors were risk factor for recurrence of HCC patients after curative treatment, such as AFP, tumor size, tumor number, pathological type, etc. (25–27). In this study, we identified only AFP levels and tumor number were associated with RFS probability in HCV-related HCC patients after thermal ablation combined with TACE. Tumor size was not risk factor for recurrence of HCC, probably because of the low proportion of patients with tumors larger than 3 cm (n=30, 36.14%).

There were some limitations in this study. First of all, this was a single-center and retrospective study, and we could not completely avoid referral bias. Second, this was a single-arm study. The effects of combined therapy on the survival of patients between HCV-related HCC and HBV-related HCC were not compared. Finally, the sample of this study is relatively small. Further prospective randomized controlled trials are necessary to validate our observations.

## Conclusions

The data of our study indicated that percutaneous thermal ablation combined with TACE is an effective and safe ablation modality for patients with HCV-related HCC. Sex, ablation result and ALBI were independent prognostic factors for survival after percutaneous thermal ablation combined with TACE for HCC.

## Data availability statement

The original contributions presented in the study are included in the article/Supplementary Material. Further inquiries can be directed to the corresponding author.

## Ethics statement

The studies involving human participants were reviewed and approved by the Ethics Committee of Capital Medical University affiliated Beijing Youan Hospital. The patients/participants provided their written informed consent to participate in this study.

## Author contributions

Conceived and designed the protocol: CY. Collected data: YS and JL. Wrote the manuscript: YS and HZ. Analyzed data: JZ and YZ. Critically revised and approved the final version of manuscript: CY. All authors contributed to the article and approved the submitted version.

## Funding

This study is supported by Capital Health Research and Development of Special Fund (No.2018-2-2182); Natural Science Foundation of Beijing Municipality (grant no. 7191004) and the Beijing Key Laboratory of Biomarkers for Infectious Diseases (grant no.BZ0373).

## Conflict of interest

The authors declare that the research was conducted in the absence of any commercial or financial relationships that could be construed as a potential conflict of interest.

## Publisher’s note

All claims expressed in this article are solely those of the authors and do not necessarily represent those of their affiliated organizations, or those of the publisher, the editors and the reviewers. Any product that may be evaluated in this article, or claim that may be made by its manufacturer, is not guaranteed or endorsed by the publisher.
